# An Experimental and Numerical Study on Glass Frit Wafer-to-Wafer Bonding

**DOI:** 10.3390/mi14010165

**Published:** 2023-01-08

**Authors:** Seyed Amir Fouad Farshchi Yazdi, Matteo Garavaglia, Aldo Ghisi, Alberto Corigliano

**Affiliations:** 1 Department of Civil and Environmental Engineering, Politecnico di Milano, Piazza Leonardo Da Vinci 32, 20133 Milano, Italy; 2 STMicroelectronics, Via Camillo Olivetti 2, 20864 Agrate Brianza, Italy

**Keywords:** glass frit bonding, wafer warpage, FE analysis, residual stresses

## Abstract

A thermo-mechanical wafer-to-wafer bonding process is studied through experiments on the glass frit material and thermo-mechanical numerical simulations to evaluate the effect of the residual stresses on the wafer warpage. To experimentally characterize the material, confocal laser profilometry and scanning electron microscopy for surface observation, energy dispersive X-ray spectroscopy for microstructural investigation, and nanoindentation and die shear tests for the evaluation of mechanical properties are used. An average effective Young’s modulus of 86.5 ± 9.5 GPa, a Poisson’s ratio of 0.19 ± 0.02, and a hardness of 5.26 ± 0.8 GPa were measured through nanoindentation for the glass frit material. The lowest nominal shear strength ranged 1.13 ÷ 1.58 MPa in the strain rate interval to 0.33 ÷ 4.99 × 10${}^{-3}$ s${}^{-1}$. To validate the thermo-mechanical model, numerical results are compared with experimental measurements of the out-of-plane displacements at the wafer surface (i.e., warpage), showing acceptable agreement.

## 1. Introduction

Among the different bonding technologies, glass frit (gf) wafer-to-wafer bonding [1,2] is well established and widely used, since it allows safely [3] and economically [4] enclosing microelectromechanical systems (MEMS) for numerous applications. It also prevents the contamination of MEMS in the cavity during the subsequent fabrication processes, such as dicing [5,6]. This technology includes screen printing of a glass paste, its thermal conditioning, and then a thermo-compressive bonding process [7]. It is important to remember that the packaging cost of an individual device can reach a significant portion of the total cost [8] and therefore efficient wafer-level packaging can save a significant amount of money. The gf bonding procedure provides hermetic sealing, a reasonable stress level at the bonding interface, high strength and reliability, together with the possibility to incorporate metallic feed throughs [2,9].

A disadvantage, however, resides in the wafer warpage due to the coefficient of thermal expansion mismatch between silicon and gf and due to other sources of residual stresses arising during the bonding process [10]. For this reason, several studies have investigated and modeled this process, with emphasis on its residual stresses and the characterization of the obtained material.

In [11], the stress induced by an excessive distribution of gf material was investigated, and its effects on the position of the zero-point of the pressure inside the inner MEMS chamber were considered. Refs. [12,13] studied the fracture toughness of a gf material by micro-chevron tests, including the influence of temperature on the toughness and on the crack path. An analysis of the bonding process during the cooling down from the glass transition temperature was treated in [14], where the authors pointed out the influence of the gf coefficient of thermal expansion, gf Young’s modulus, bonding temperature, and gf ring thickness on the wafer warping. Less important parameters were also identified into the ring width and cover wafer thickness. In [15], however, the width and height of the gf bonding layer influenced the bonding strength and hermeticity, and a technique to carefully control these geometrical dimensions was proposed. In [16], again by means of micro-chevron tests, the requirement to carefully investigate the integrity of the bond interface during the experiments was demonstrated via scanning acoustic microscopy. In the absence of the knowledge about the effective bonding geometry (i.e., as obtained from process), wrong conclusions would be inferred from the tests. For [17], lead precipitates in the gf layer can reduce the strength at the gf-Si interconnection during tensile tests, while their influence appeared lower in micro-chevron tests. Ref. [18] tackled a problem similar to [11] and showed the influence of the gf material distribution on the zero point for a piezoresistive pressure sensor by means of finite element (FE) numerical simulations. They also applied thermal cycles to the gf material after bonding, finding an increase of the microcracks at the outer perimeter of excess gf.

Gf distribution can influence the MEMS sensor behaviour, as shown in [18]. The sensor zero point was affected by the residual stresses in the gf layer and, in particular, by the gf excess material along the seal frame perimeter. The gf layer was also possibly affected by microcracks in the excess material region after thermal cycling, explaining the shifts experienced in the sensor output signal. Understandably, strategies to improve the uniformity of the gf bonding layer were studied in the past, see. e.g., [19], and also, more recently, a silicon wet etching technique to control gf bonding height and width was proposed in [15].

Gf bonding, together with all the other deposition steps, can be still delicate, as it was also recently shown in [20], where voids of unclear origin were observed in a batch of isolation gf trenches.

In most of the cases mentioned above, a significant standard deviation was found for the strength and fracture results in view of the non-homogeneous gf interface, see, e.g., [21].

Gf is nowadays also adopted to seal other ceramic materials, see, e.g., [22,23], and, in its lead-free version or in a mix with other materials such as graphene, for solar cells [24]; however, here we focus on its traditional application in the microsystem industry [20,25], and we aim to carefully characterize the material as obtained from the foundry, so that the subsequent calculations regarding wafer warpage can be more accurate.

In this paper, first, in the next Section 2, a characterization of gf with the aim of obtaining the effective elastic parameters in wafer-to-wafer bonding was pursued; then, shear tests at varying strain rates were considered to measure the interface bonding strength. For these experiments, closed dies were used, as obtained from the actual bonding process. Finally, the complexity of the stress and temperature changes during the bonding process is addressed by a three-dimensional finite element model described in Section 3, and the wafer out-of-plane displacements at the end of the process are compared with the experimental measurements.

## 2. Glass Frit Experimental Characterization

In gf bonding, silicon wafers are sealed through a thermo-mechanical process by exploiting low melting point glasses, i.e., lead or lead-silicate glasses, as an intermediate layer. The glass transformation into the viscous paste is critical in this method; hence, there are criteria to obtain a high bonding quality, e.g., controlling the grain size [7]. The knowledge of gf properties and its thermo-mechanical behaviour are of paramount importance for the modelling, simulation, and critical understanding of the bonding process which leads to residual stresses and wafer warpage. The gf material used in this research is a lead–silicate glass (DL11-036) made by Ferro [26]. Oxides contained in the glass are reported in Table 1.

Since by combining with the process, the material composition influences the mechanical behavior, Section 2.3 reports the gf microstructural study through scanning electron microscopy (SEM) and energy dispersive X-ray spectroscopy (EDX). The filler also has the role of reducing the coefficient of thermal expansion (CTE), which in this case was equal to $9\times 10^{-6}$ °C${}^{-1}$, as reported in the material data sheet [26].

There are two sets of specimens used in this research: (i) the gf paste on silicon wafer after thermal conditioning, hereafter known as *pre-bond*; (ii) silicon die after gf bonding and dicing with Si–gf-Si structure (whose thicknesses are 725, 20 and 725 μm, respectively), hereafter known as post-bond. The specimens were tested by adopting various experimental methods; in this Section, the obtained results are reported and critically discussed.

### 2.1. Confocal Laser Profilometry

**Figure 1:**
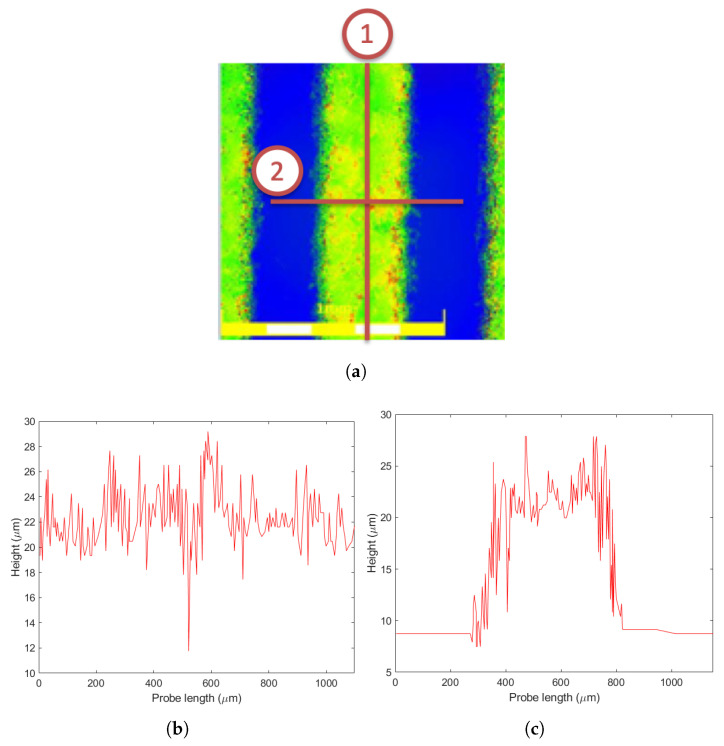
Confocal laser profilometry scanning paths and corresponding height profiles: (**a**) probe paths in the laser profilometry test (Path 1: longitudinal, Path 2: transversal); (**b**) profile from longitudinal Path 1; (**c**) profile from transversal Path 2.

Surface roughness plays a secondary role in gf wafer level packaging because during the thermo-compressive bonding the glass reaches a wetting temperature causing the material to become soft enough to flow and fuse with the wafer surface layer at the atomic level; after cooling, the obtained bond can be quite strong. The material retains a low viscosity in order to avoid flooding the cavity where the MEMS is placed. Therefore, it is the temperature more than the pressure that is important, the former in the range 425–450 °C, the latter necessary to deal with a possible wafer bow and warp. However, in the initial temperature rise, the wafers are put into contact with the gf in between by applying a mechanical pressure in addition to the partial glass melting; hence, the gaps induced by the roughness between the gf film and the silicon wafer are compensated if they are not excessive. To study the topology of gf film as well as its surface roughness, confocal laser profilometry tests were carried out on pre-bond specimens. In this test, two different probe paths, longitudinal and transversal, as depicted in Figure 1a, were selected. The longitudinal profile travelled on the gf layer surface and determined the mean height of the asperities, while the transversal profile measured the height with respect to the silicon surface, considered as the reference plane. The corresponding height profiles for each path are presented in Figure 1b,c. The surface roughness parameters, in addition to the topological parameter of the gf film obtained from the profiles, are reported in Table 2.

**Figure 2:**
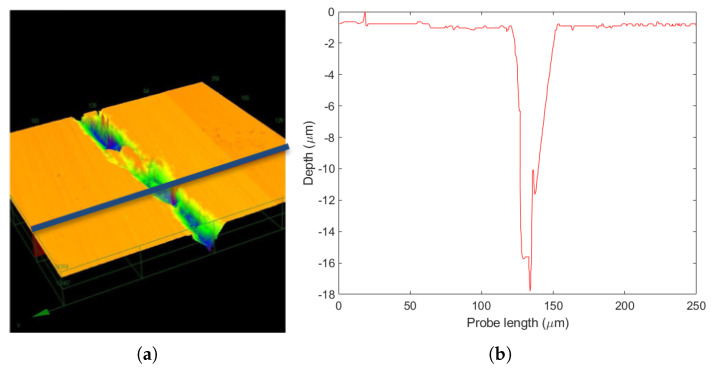
Laser profilometry on a Si−gf−Si structure: (**a**) laser probe path; (**b**) height profile.

Another set of tests was carried out for post-bond specimens along the thickness on a silicon die (with the Si–gf–Si structure mentioned above). By performing the profilometry on the cross section of this structure (blue line in Figure 2a), a difference of about 17 μm was observed in the gf region (Figure 2b). This valley-shaped profile showed that the gf thermal expansion coefficient was significantly higher than the silicon one, thus leading to a larger gf contraction in the bonding cooling phase with respect to the silicon.

### 2.2. Nanoindentation Tests

**Figure 3:**
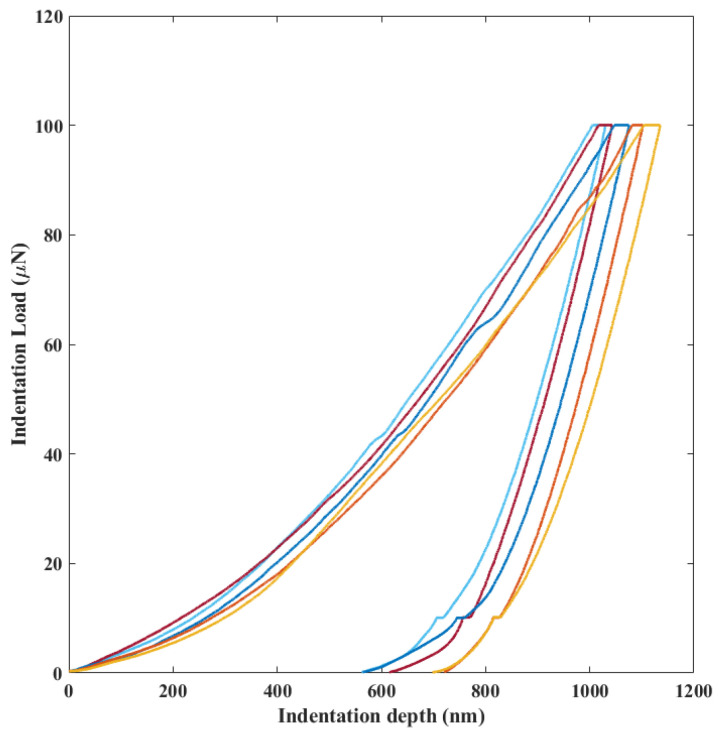
Exemplary indentation load-depth curves to obtain the elastic mechanical properties of the gf film.

To estimate the effective elastic mechanical behaviour for the considered bonding material, a Berkovich nanoindenter was used at room temperature on gf strips deposited on a silicon wafer substrate (pre-bond specimens). Two sets of measurements were carried out, one at the centre of the wafer and the other near the wafer edge. To avoid the scattering due to the surface roughness compared to the indenter radius, for each position on the wafer a set of measurements was carried out: 17 tests were performed at 100 μm intervals along the axis of the strip; then, the procedure was repeated along two additional lines, parallel to the axis but at an offset of ±100 μm, for a total of 51 measurements at each position (i.e., centre, periphery) on the wafer. Figure 3 shows instances of the force-depth curves of the nano-indentations tests. As shown in Table 3, no significant changes were observed between the results obtained at the centre and those obtained at the periphery (i.e., near the wafer edge) for the effective elastic properties at room temperature. An average (including both centre and peripheral positions) effective Young’s modulus of 86.5 ± 9.5 GPa, a Poisson’s ratio of 0.19 ± 0.02, and a hardness of 5.26 ± 0.8 GPa were therefore measured.

Since gf mechanical properties are highly dependent on the composition, different values are reported in the literature. For instance, Ebert and Bagdahn [27] indicated *E* = 50 GPa and ν = 0.23 for Young’s modulus and Poisson’s ratio, respectively. Grabham et al. [28] inspected the mechanical properties of gf binder material through experiments on cantilever beams loaded at the free ends and numerical simulations. Different gf materials were deposited on a silicon substrate; the authors obtained an effective Young’s modulus for two gf pastes equal to 22 GPa and 33.5 GPa, respectively, but they commented that these values were more indicative of the aggregate than of the actual gf material. Dresbach et al. [12] reported instead E=55 GPa and ν=0.23; Xu et al. [14] investigated the effect of Young’s modulus on the final warpage of the gf bonding, observing that the higher the Young’s modulus, the higher the final warpage: the material in the present study, therefore, should emphasize the warping effect.

### 2.3. Microstructural Study

**Figure 4:**
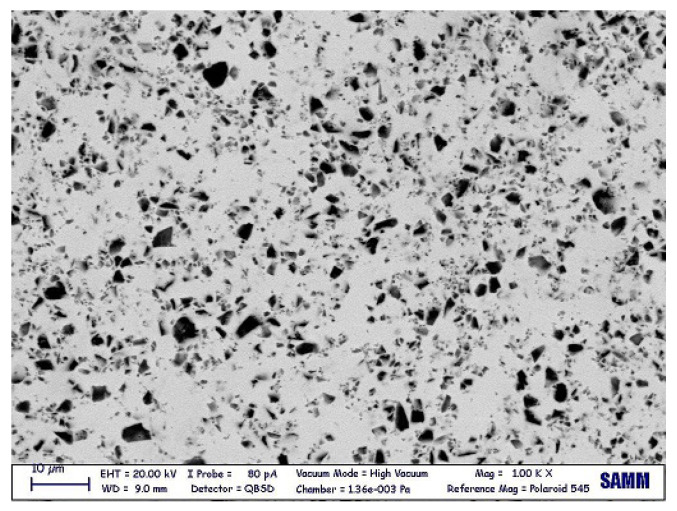
Gf microstructure, as observed via a SEM.

A SEM observation was carried out to characterize the microstructural features of the gf film. The image, obtained in backscattered electron beam mode, is shown in Figure 4 and demonstrates the dual-phase morphology, in the sense of distribution of a darker filler (Al${}_{2}$O${}_{3}$) in a brighter matrix (PbO). Initially, the filler area ratio was calculated by image processing, obtaining 17.3%.

**Figure 5:**
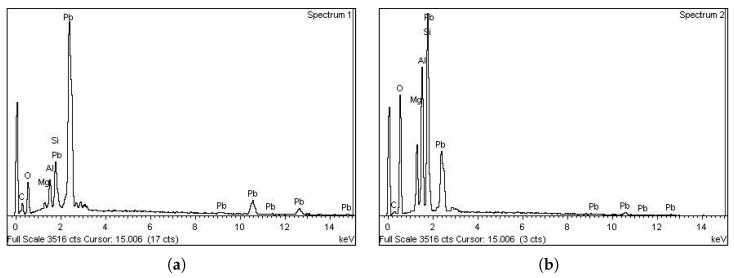
EDX spectroscopy analysis: (**a**) EDX spectrum for the matrix; (**b**) EDX spectrum for the secondary phase.

Next, for a deeper study, EDX was performed on each phase to obtain the chemical components. Based on the results of this test, shown in Figure 5, the chemical composition of the matrix appeared to be lead, while cordierite ((Mg,Fe)${}_{2}$Al${}_{3}$(Si${}_{5}$AlO${}_{18}$)) was detected as the secondary phase.

Notable remarks related to gf are the following: by increasing the amount of lead in lead silicate glass, the hardness of the material decreases [29]; the kinetics of phase separation and inter-phase atomic diffusion in lead-silicate glasses starts at 500 °C [30]. Hence, during the bonding process, where the maximum temperature is below the aforementioned value at about 440 °C, the phase ratio and distribution will not change.

### 2.4. Die Shear Test

**Figure 6:**
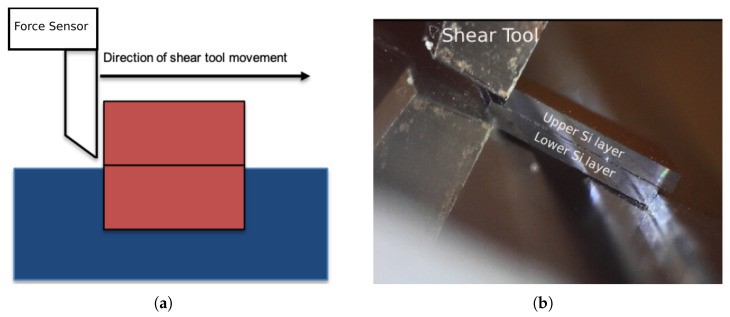
Die shear test setup: (**a**) die shear test working principle; (**b**) shear test inspection by usb microscope.

The mechanical strength and reliability of silicon dies bonded by gf were measured by die shear tests, according to the standard MIL-STD-883E [31]. In these tests, for a single closed and naked die, the upper silicon cap was displaced by a metallic tool, while the silicon substrate was held in place by a convenient grip (Figure 6a); a Condor Sigma Lite machine by XYZTEC was used. During the tests, the tool displacement was imposed and the reaction force, as obtained by the force sensor at the tool itself, was measured. In the standard, the failure load determines the sufficient bonding strength with respect to the die size; in this work the test was carried out at different displacement rates (1, 5, 10, and 15 μm/s) to study the gf behaviour. In total, 65 post bond specimens were tested. To control and investigate the detachment behaviour under shear load, the tests were observed by a DinoLite USB microscope as shown in Figure 6b, which could magnify the image up to 140% in the long focal distance.

The results of the die shear tests could be categorized into three groups based on the load-displacement curves and the detachment behavior of the upper silicon layer.

**Figure 7:**
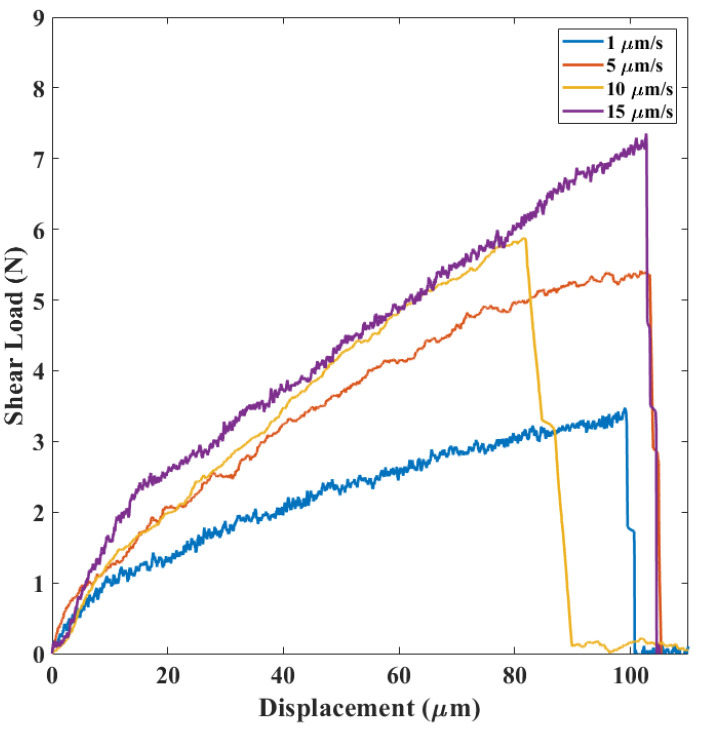
Load-displacement results in category A.

In category A, see Figure 7, a nonlinear relationship between shear load and displacement with progressive stiffness reduction was observed. Moreover, after completion of debonding, the upper part of the silicon die was displaced in the direction of the load applied by the shear tool.

**Figure 8:**
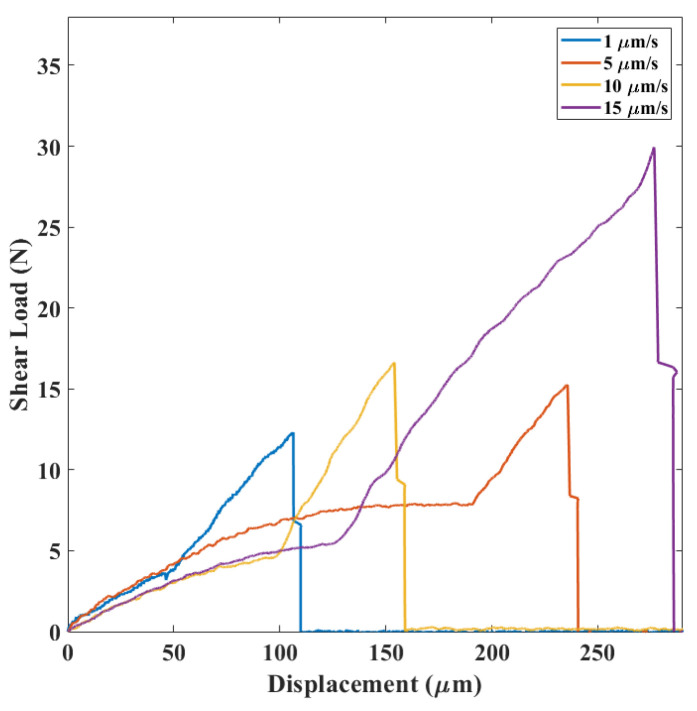
Load-displacement results in category B.

In category B, see Figure 8, a sudden increase of stiffness was observed after a behavior similar to the first case. The upper part of the silicon die experienced a rapid displacement in the load direction and normal to it.

**Figure 9:**
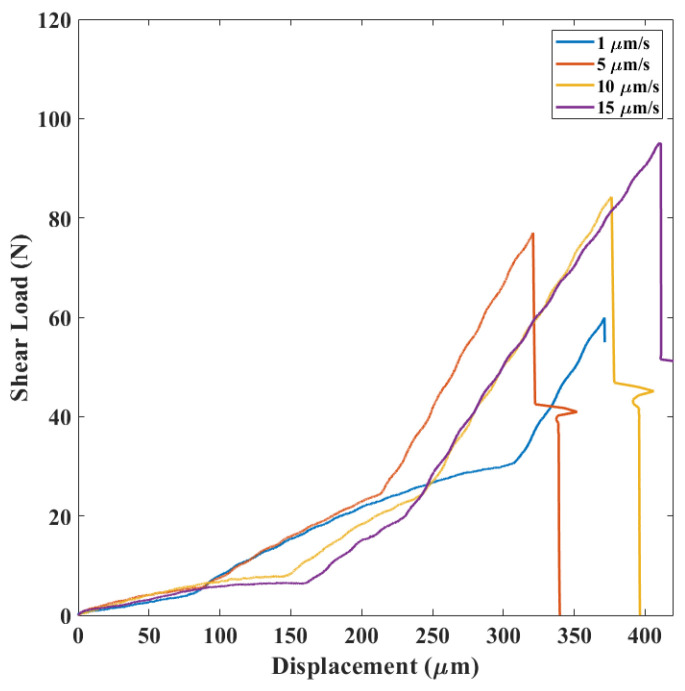
Load-displacement results in category C.

In category C, see Figure 9, a three-part curve was obtained. Crack initiation and propagation within the silicon layer was noticed in these cases.

**Figure 10:**
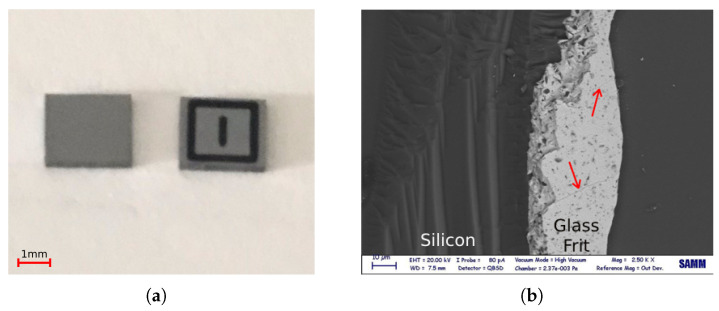
Optical observations after shear test, category A: (**a**) Si die parts after debonding; (**b**) cracks on gf surface.

In category A, after die failure, the gf layer remained almost intact, as shown in Figure 10a. Furthermore, cracks were detected on the fracture surface of gf via SEM, see Figure 10b. These implied that in this category mode-II fracture (pure shear mode) occurred at the Si–gf interface.

**Figure 11:**
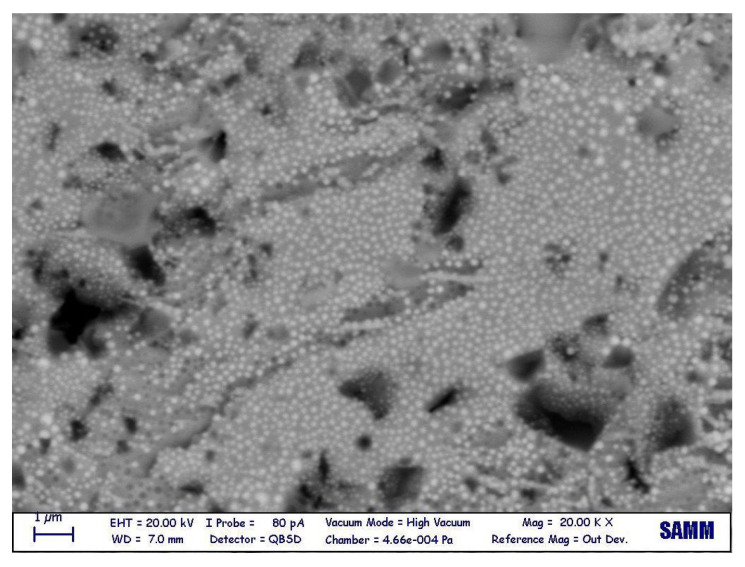
Spherical lead particles on the glass fit fracture surface after the die shear test.

Spherical lead particles were also observable on the fracture surface after debonding, see Figure 11. The same appearance was reported for the fracture surface after gf debonding in the mode-I fracture (opening mode) by Dresbach et al. [12].

**Figure 12:**
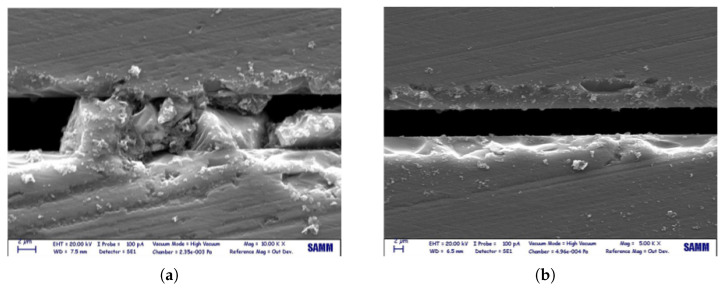
Effect of dicing process on the silicon layers (die cross-section view): (**a**) silicon debris at the interspace of the silicon layers; (**b**) defects introduced by dicing saw.

The failure mechanisms for the categories B and C stem most probably from the dicing as a subsequent step after thermo-mechanical bonding during the microsystem fabrication. Dicing left silicon debris at the interspace between silicon layers in the die; moreover, it introduced microcracks and defects in the silicon layer, where material is mechanically susceptible because of the higher stress intensity. Figure 12 shows dicing effects on a single die bonded with gf.

**Figure 13:**
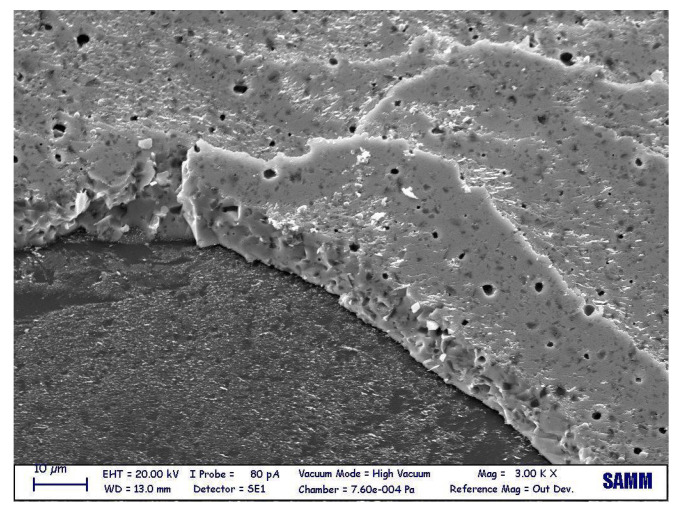
SEM image of the gf layer after the die shear test failed in the mixed-mode mechanism.

In failure category B, the presence of silicon debris led to deviation of the effective applied load from the tangential to the normal direction with respect to the gf plane. The silicon layer experienced a projectile motion after debonding. Moreover, Figure 13, which is a SEM image of the fracture surface failure in this category, shows a crack propagation perpendicular to the gf layer. It was concluded that the failure mechanism corresponding to this behaviour was a mix of mode-I and mode-II fracture, and the additional linear part of the load-displacement curve with respect to category A was originated from an additional mode-I fracture opening.

**Figure 14:**
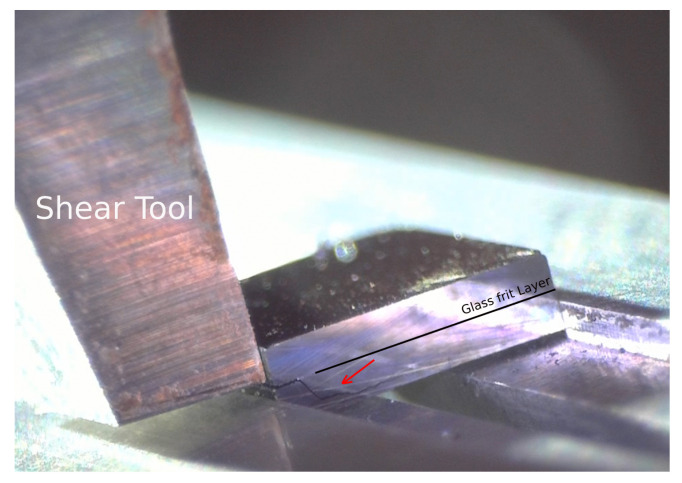
Crack propagation in the silicon layer for category C as captured by a DinoLite microscope. The arrow points to the crack path.

In category C, the die failed because of a silicon rupture, most probably by propagation of the cracks initiated at the defects of the pre-existing microcracks brought in by the dicing saw and then deviated into the silicon layer (see Figure 14). Since the silicon layer mechanical strength is significantly higher than the gf layer’s strength, the peak load was higher among all failure mechanisms.

**Figure 15:**
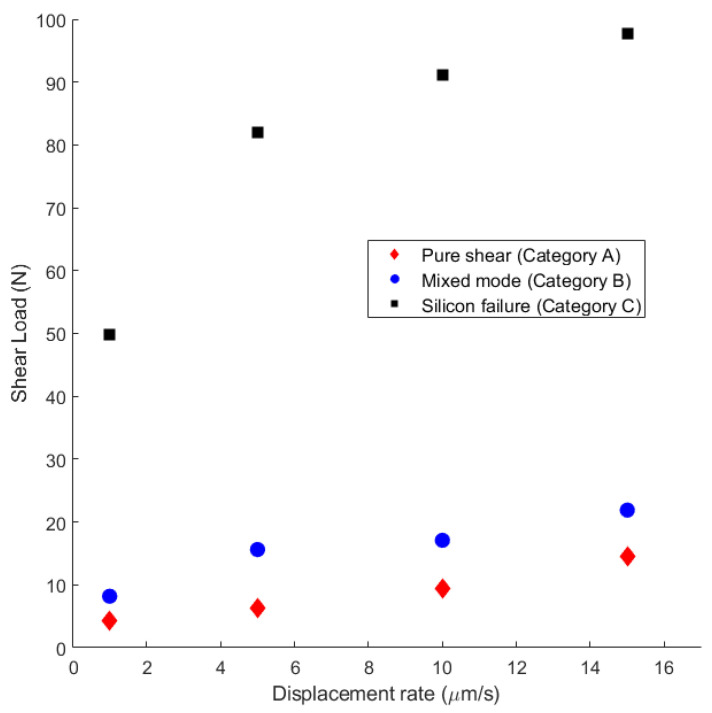
Dependency of the failure load with respect to the strain rate.

By studying the relationship between the average shear load and the displacement rate for each mechanism (Figure 15) at different applied displacement rates in the range 1–15 μm/s, all the failure mechanisms were observed. Second, regardless of the mechanism, the higher the displacement rate, the higher the failure load was. Moreover, by comparing the load-displacement curves for different failure mechanisms (Figure 7, Figure 8 and Figure 9), the slope of each region was constant at different strain rates, indicating that each region belonged to a specific failure mechanism.

It can be concluded that there was a competition between each mechanism, and depending on the condition of the die under the test, the failure occurred following a specific mechanism.

**Figure 16:**
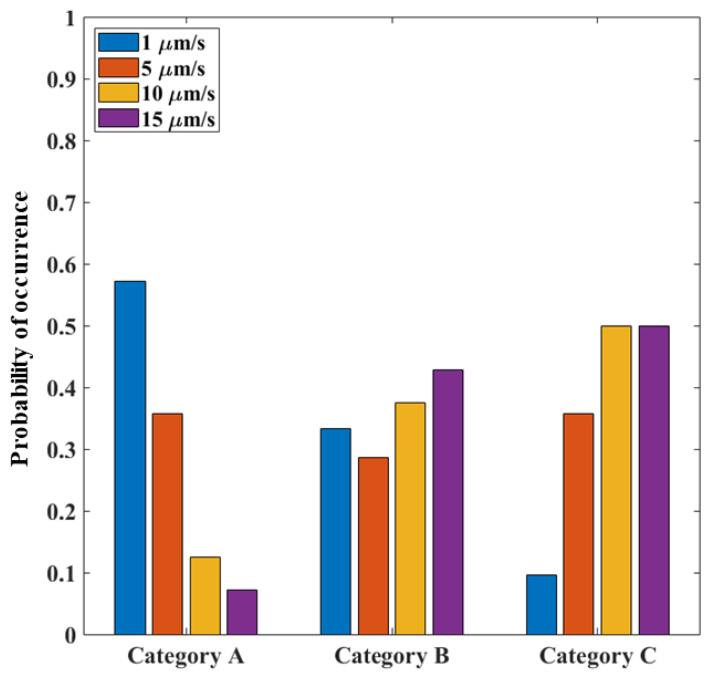
Probability of mechanism occurrence at different strain rates.

To investigate the effect of strain rate on the probability of occurrence related to a failure category, the percentage of each failure mechanism at a given strain rate was calculated and shown in Figure 16. The results showed that the category A (pure mode-II failure) was highly probable at the lower displacement rates, since the lower rate was not high enough for the dislocation of the debris or the creation of cracks in silicon, and the die failed at the Si−gf interface.

Category C (silicon rupture) mainly occurred instead at high strain rates due to the vulnerability to defects at higher strain rates.

For the mixed mode mechanism related to category B, the probability was found at similar values for all the applied displacement rates because of the presence of silicon debris at the silicon layer interstices with randomly distributed sizes.

Both for category B and C, the load was higher than for category A because the crack path diverged from the purely horizontal (shear-induced) direction and started deviating slightly inside the gf layer itself or inside the silicon layers; globally, the resistance appeared to increase. This behaviour confirmed the good quality of the attained gf bonding.

In view of all the considerations above, category B and C responses could not be included in the interface strength characterization because of the mode mixity and of the crack propagation variability. Table 4, Table 5 and Table 6 show the nominal shear strength (defined as the horizontal force over gf area) for different failure mechanisms. It should be emphasised that since the crack surface in categories B and C deviated from the Si–gf interface and because of the complete rupture (i.e., destruction) of the specimen (in category C), there was no possibility to measure the fracture surface; therefore, the reader should understand that the true shear stress for these mechanisms would be different. As presented in Figure 6, failure by pure shear mode (category A) was a subset with respect to the two other failure types, and it could not be strictly considered as the die strength., However, it only represented the strength of the Si–gf interface in the mode-II fracture, i.e., a lower bound.

## 3. Numerical Analysis

**Figure 17:**
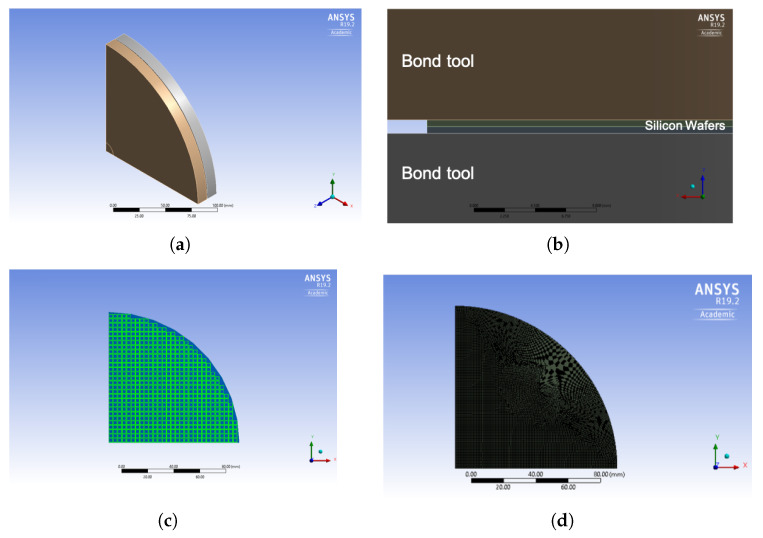
Finite element model of gf bonding: (**a**) one-quarter of the bonding chamber; (**b**) planar view of the FE model; (**c**) gf region; (**d**) FE model mesh.

The thermo-mechanical wafer-to-wafer bonding process was modelled through a three-dimensional FE model. As a first approximation exploiting double symmetry, one-quarter of the components inside the bonding chamber was considered in the FE model, see Figure 17a. Upper and lower wafers were constrained with a unilateral frictionless contact through two bond tools by which the mechanical constraints as well as the heat flux conditions were applied to the 8-inch silicon wafers; gf lines (with 3 mm length and 0.5 mm width) were placed at die borders, as shown in Figure 17b and Figure 17c, respectively.

The bonding process consists of a multi-step temperature profile similar to the one shown in [4] with a simultaneously applied mechanical pressure in an environment encompassing some hundreds of millibars of nitrogen gas. The wafers were held at the maximum temperature for 5–10 min, and subsequently, the wafers were cooled down. As a final step, the mechanical pressure was removed, and bonded wafers were taken out from the chamber to reach the ambient temperature.

**Figure 18:**
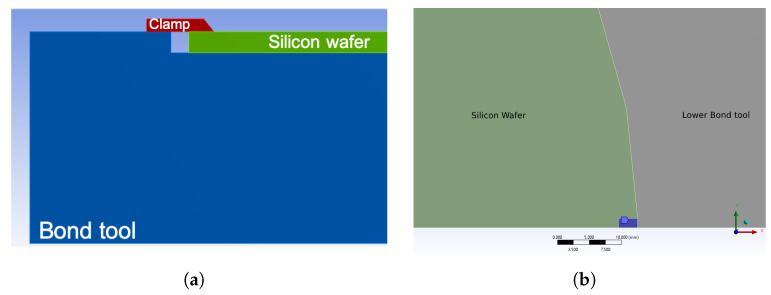
Bond tool clamps: (**a**) bond tool clamp configuration on the wafer; (**b**) zero degree of freedom area (blue) in the FE model (x-y plane).

The simulation of the bonding process followed a decoupled approach. First, a thermal analysis was carried out and the temperature distribution within the wafer was obtained during the evolution from the initial value to the final bonding temperature imposed on the chamber (between 400 ${}^{{}^{\circ}}$C to 440 ${}^{{}^{\circ}}$C) and then during the decrease back to the initial state. The time-dependent temperature profile was applied at the surface at both bond tools. In the second step, a mechanical load (about 10 kN) was imposed to the upper bond tool against the lower substrate wafer in addition to the temperature increment histories, as imported from the thermal analysis carried out in the first step. As depicted in Figure 18a, silicon wafers were mechanically clamped to the bond tools: a mechanical constraint was imposed in this area in terms of zero displacements and rotations (Figure 18b). Due to the simplification, the nitrogen hydrostatic pressure was neglected.

The aim of the numerical simulation was to estimate the warpage in terms of the out-of-plane displacements at the wafer upper surface. Moreover, the stress distribution during and at the end of the bonding process was also studied.

### 3.1. Materials and Modelling Details

Eight-inch (100) mono-crystalline silicon wafers were considered. An orthotropic elastic behaviour was considered for silicon, and the stiffness coefficients were assigned with respect to the directions of the axes of elasticity, according to [32]. The bond tool for the lower wafer was made of titanium and the upper one was made of stainless steel. They were constrained to the wafers by unilateral contact, imposed through the Lagrangian multiplier method neglecting friction; the contact allowed perfect heat transfer. Moreover, the lateral constraining components transferred the thermal flux to the silicon wafers. The thermal and mechanical properties for these parts are described in Table 7 and Table 8. Since a coupled-field thermo-mechanical analysis was performed, the mechanical properties were considered temperature-dependent.

The temperature dependency of silicon elastic stiffness can be expressed as below:

(1) $$C_{ij}\left(T\right)=C_{ij}\left(T_{0}\right)\;\left[1+\sum_{k\geq 1}TCE{\left(C_{ij}\right)}_{k}\;{\left(T-T_{0}\right)}^{k}\right]$$

where $C_{ij}$ is a generic stiffness coefficient, *T* is the expected temperature, $T_{0}$ is the reference temperature, *k* is the order of the coefficients, and $TCE(C_{ij})$ is the temperature coefficient reported by Bourgeois et al. [33]. For the other materials used in this model, i.e., titanium and stainless steel, the temperature dependency of their elastic properties was obtained from [34,35], respectively.

Interface FEs [36], traditionally employed in computational mechanics for delamination or fracture processes, were exploited to model the gf layer. This choice was motivated by the small thickness of the gf material interconnecting the two silicon wafers with respect to the silicon wafers: a fully three-dimensional model would have generated too large a number of FEs and too many degrees of freedom. In interface elements, the constitutive material behaviour is defined through a cohesive zone approach, linking the tractions at the interface to the displacement discontinuity vector; most importantly for the present application, their topology did not require other nodes with respect to the ones at the two facing surfaces. A bilinear traction-displacement discontinuity law, linearly increasing up to the maximum strength and then linearly decreasing down to zero, defined the behaviour of the interface element [37]. In the linearly increasing branch, the equation of the cohesive material can be expressed as follows:

(2) $$T_{i}=K_{i}\delta_{i}$$

$K_{i}$ being the cohesive stiffness, $T_{i}$ the cohesive traction (in normal and tangential direction), $\delta_{i}$ the displacement discontinuity at the interface (in normal and tangential direction), and the subscript *i* denoting the normal and the tangential directions with respect to the gf plane. The normal stiffness was assigned according to the study by Dresbach et al. [12]; the tangential stiffness was assigned to gf according to the results obtained by the die shear tests described in Section 2.4.

The bonding interface formation in the model was considered in terms of the variation of $K_{i}$ for the interface FEs with respect to the temperature. Before reaching the glass transition temperature, the stiffness had a very low value; after reaching the highest point in the temperature profile $K_{i}$ values were changed, namely the value reported by Dresbach et al. [12] was assigned to the stiffness in the normal direction, while for the stiffnesses along the tangential direction, the linearized stiffness obtained from the die shear test (see Section 2.4) was assumed. Interface stiffness values remained constant in the cooling phase.

The calculations were carried out with the commercial FE analysis code ANSYS 19.2. At each step, the transient thermal analysis preceded the mechanical calculation. Besides the mentioned bond tools, the bottom surface of the lower plate was simply supported, while the top surface was loaded by a pressure whose resultant was equal to 10 kN, as mentioned above. The temperature history was imposed at the same top and bottom surfaces. The mesh of the whole three-dimensional model encompassed about 100,000 nodes and 320,000 cubic quadratic elements (SOLID186), see Figure 17d.

### 3.2. Numerical Results

The thermal analysis confirmed a uniform temperature distribution inside the bonding chamber, as was expected from the small thickness of the wafers and also because the temperature was held constant long enough at each process step. From the analysis of the temperature profile along the length of the silicon wafer at different bonding temperatures, no thermal stresses were expected within each layer during the bonding process.

**Figure 19:**
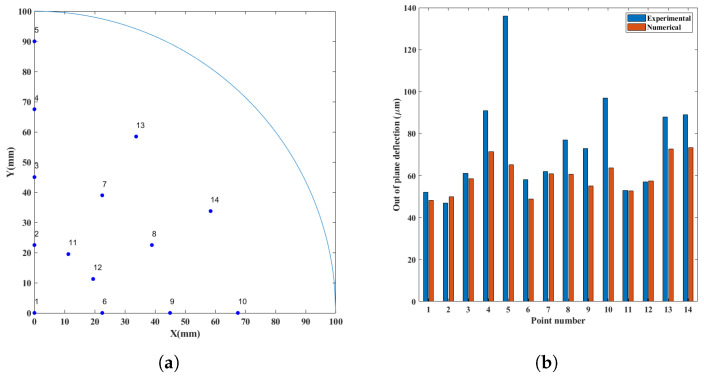
Comparison of numerical and experimental out-of-plane displacements: (**a**) point coordinates on the wafer surface; (**b**) out-of-plane displacements.

**Figure 20:**
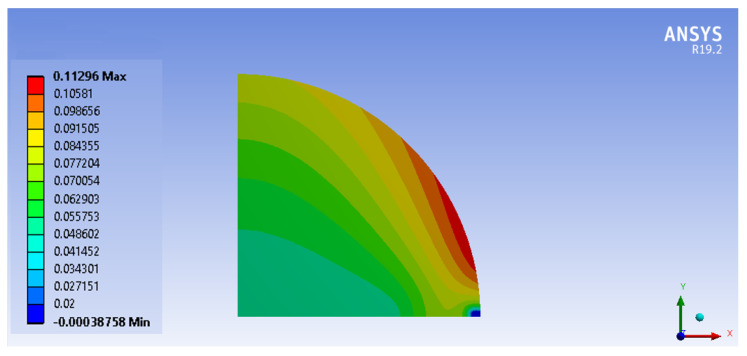
Out-of-plane displacements (in millimeters) after a complete thermo-mechanical bonding cycle.

The mechanical simulation showed a final convex shape that was compared with the experimental observations at 14 points on the wafer surface. The comparison is shown through histograms in Figure 19. The convex shape seems correctly reproduced, even if slightly underestimated. The maximum simulated deflection (i.e., out-of-plane displacement) was 112 μm, see Figure 20.

**Figure 21:**
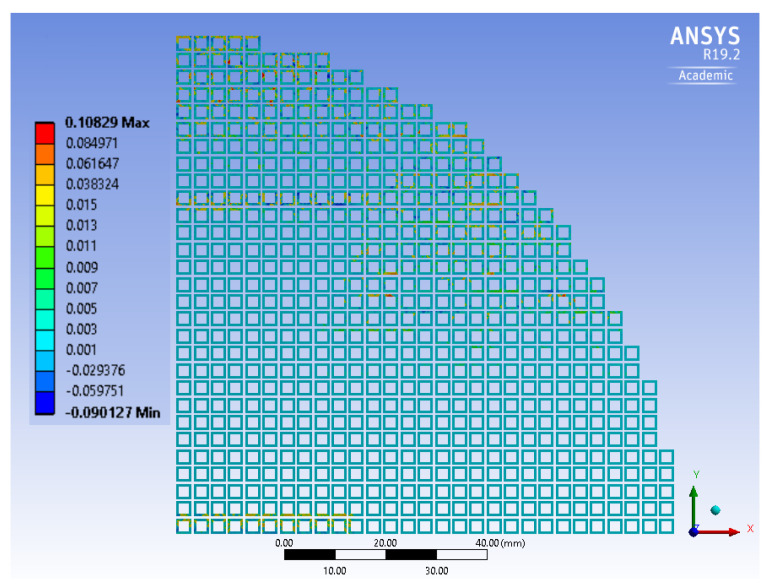
Tangential traction for interface elements (in MPa).

Moreover, Figure 21 shows the tangential traction at the end of a complete bonding cycle at the gf region. The maximum value for this parameter reached 0.1 MPa, which was significantly lower than the gf interface debonding stress reported in Table 4.

The difference between the numerical and experimental results could be due to the following reasons: (i) the traction-displacement jump in the model was considered linear, while in the die shear test it was found nonlinear, (ii) the gf stiffness was considered invariant in the cooling phase, and (iii) the silicon CTE was assumed isotropic, while it is anisotropic in reality. Nevertheless, in view of these compromises, the outcome still showed a pronounced improvement with respect to the existing models in the literature, e.g., [38].

## 4. Conclusions

This work focused on glass frit, a diffused bonding material in the MEMS industry. Experimental results were used to calibrate numerical simulations to estimate the effects of residual stresses arising during the thermo-compressive bonding. It was shown that:

- The gf material was homogeneously deposited on the wafer since its effective elastic properties did not vary between wafer centre and periphery, as shown by the nanoindentation tests;
- A rather large scattering of the nominal shear strength was observed in the shear tests due to the role of the defects; however, the crack path often involved the bulk silicon in the die, thus confirming the good bonding quality;
- Stiffnesses and strength, as obtained from the shear tests, were adopted to model the gf layer during the thermo-compressive process via cohesive elements, allowing a three-dimensional numerical simulation; even with some simplifications (discussed at the end of Section 3.2), the comparison between simulated and observed out-of-plane displacements at the wafer surface appeared acceptable.

It is worth noting that it is the combination of an experimental and numerical approach that allows us to write the conclusions mentioned above, and this work plan will also be followed for future developments. In view of the results, some variants to the process can be studied, such as [39], and improvements to the numerical modelling could be envisaged; in particular, gf temperature-variant properties, that in turn need to be experimentally quantified, could be included in the simulation.

## Figures and Tables

**Table 1:** Oxide content as a percentage for the gf material.

Oxide	Composition, Mean Percentage (%)
SiO${}_{2}$	10 ± 3
Al${}_{2}$O${}_{3}$	6 ± 3
MgO	1.8 ± 0.5
CaO	0.1 ± 0.05
BaO	0.3 ± 0.1
PbO	66 ± 4
B${}_{2}$O${}_{3}$	13 ± 3
ZnO	2.6 ± 0.5
Na${}_{2}$O	0.2 ± 0.1

**Table 2:** Topological and surface roughness parameters of the gf film.

Parameter	Value
Arithmetic average height ($R_{a}$)	1.585 μm
Mean square root height ($R_{q}$)	2.168 μm
Film height ($R_{t}$)	20.136 μm

**Table 3:** Effective elastic properties and hardness for the gf material, as obtained from nanoindentation tests at room temperature.

Position in the Wafer	Young’s Modulus (GPa)	Poisson’s Ratio	Hardness (GPa)
Centre	87.54 ± 9.34	0.19 ± 0.02	5.35 ± 0.85
Peripheral	85.55 ± 9.79	0.19 ± 0.02	5.17 ± 0.74

**Table 4:** Nominal shear stress at different strain rates, category A.

Strain Rate (1/s)	Mean Shear Stress (MPa)
0.33 × 10${}^{-3}$	1.13 ± 0.32
1.66 × 10${}^{-3}$	1.14 ± 0.24
3.33 × 10${}^{-3}$	1.42 ± 0.17
4.99 × 10${}^{-3}$	1.58 ± 0.26

**Table 5:** Nominal shear stress at different strain rates, category B.

Strain Rate (1/s)	Mean Shear Stress (MPa)
0.33 × 10${}^{-3}$	2.55 ± 1.05
1.66 × 10${}^{-3}$	2.65 ± 0.46
3.33 × 10${}^{-3}$	2.79 ± 0.70
4.99 × 10${}^{-3}$	4.02 ± 1.02

**Table 6:** Nominal shear stress at different strain rates, category C.

Strain Rate (1/s)	Mean Shear Stress (MPa)
0.33 × 10${}^{-3}$	12.44 ± 3.74
1.66 × 10${}^{-3}$	12.65 ± 3.49
3.33 × 10${}^{-3}$	12.67 ± 4.34
4.99 × 10${}^{-3}$	12.73 ± 2.13

**Table 7:** Mechanical properties assigned in the model at room temperature.

Material	Elastic Moduli (GPa)	Poisson’s Ratio
Silicon	E${}_{x}$ = E${}_{y}$ = 169	$\nu_{xy}$ = 0.064
	E${}_{z}$ = 130	$\nu_{yz}$ = 0.36
	G${}_{xy}$ = 50.9 G${}_{xz}$ = 79.6	$\nu_{xz}$ = 0.28
Titanium	96	0.36
Stainless steel	193	0.31

**Table 8:** Thermo-mechanical properties assigned in the model at room temperature.

Material	Thermal Conductivity (W·m${}^{-2}\cdot$ °C${}^{-1}$)	CTE (×10${}^{-6}$ °C${}^{-1}$)
Silicon	124	2.46
Titanium	21.9	9.4
Stainless steel	15.1	1.7

## Data Availability

Samples of the broken specimens are available from the authors.
